# A study of GJB2 and delGJB6-D13S1830 mutations in Brazilian non-syndromic deaf children from the Amazon region

**DOI:** 10.5935/1808-8694.20130016

**Published:** 2015-10-14

**Authors:** Luciana Santos Serrão de Castro, Anderson Nonato do Rosario Marinho, Elzemar Martins Ribeiro Rodrigues, Giorgio Christie Tavares Marques, Tarcísio André Amorim de Carvalho, Luiz Carlos Santana da Silva, Sidney Emanuel Batista dos Santos

**Affiliations:** PhD in Genetcs and Molecular Biology (Researcher); PhD in Genetcs and Molecular Biology (Researcher); Degree in Biomedicine; MSc in Genetcs and Molecular Biology (Researcher); PhD (Associate Professor I - Institute of Biological Sciences - Federal University of Pará); PhD (Associate Professor III - Institute of Biological Sciences - Federal University of Pará). Institute of Biological Sciences - Federal University of Pará

**Keywords:** connexins, deafness, genetic counseling

## Abstract

Hearing impairment affects about 1 in 1000 newborns. Mutations in the connexin 26 (GJB2) gene rank among the most frequent causes of non-syndromic deafness in different populations, while delGJB6-D13S1830 mutation located in the DFNB30 locus is known to cause sensorineural hearing loss. Despite the many studies on the involvement of GJB2 mutations in hearing impairment in different populations, there is little information on genetic deafness in Brazil, especially in the Amazon region.

**Objective:**

To determine the prevalence of GJB2 mutations and delGJB6-D13S1830 in 77 sporadic non-syndromic deaf patients.

**Method:**

The coding region of the GJB2 gene was sequenced and polymerase chain reaction was performed to detect the delGJB6-D13S1830 mutation.

**Results:**

Mutant allele 35delG was found in 9% of the patients (7/77). Mutations M34T and V95M were detected in two distinct heterozygous patients. Non-pathogenic mutation V27I was detected in 28.6% of the patients (22/77). None of the deaf patients carried the delGJB6-D13S1830 mutation.

**Conclusion:**

Mutant alleles on gene GJB2 were observed in 40% (31/77) of the subjects in the sample. Pathogenic variants were detected in only 12% (9/77) of the individuals. More studies are required to elucidate the genetic causes of hearing loss in miscegenated populations.

## INTRODUCTION

Hearing impairment affects about 1 in 1000 new-borns^1^. Deafness can be due to either genetic or environmental causes or a combination of both. In developed countries, about 60% of all cases are related to genetic origin^2^. It has been estimated that 30% of genetic deafness cases are syndromic and 70% are non-syndromic^3^. In Brazil, the frequency of non-syndromic congenital deafness is approximately four in 1000 births, 16% of which are of genetic etiology^4^. Within the hereditary non-syndromic hearing loss category, autosomal recessive forms (DFNB) make up about 75-80% of all cases, autosomal dominant forms (DFNA) about 20%, X-linked forms (DFN) 2-5%, and mitochondrial forms about 1%^5^. To date, more than 120 different loci have been estimated to be involved in deafness and 70 genes have been identified and characterized^6^.

The *GJB2* locus has been reported to be the major cause of autosomal recessive non-syndromic sensorineural deafness (ARNSD)^3^^,^^7^^,^^8^. *GJB2* gene mutations have been associated to 50% of autosomal recessive non-syndromic deafness in many populations^9^^,^^10^. More than 101 different mutations in connexin 26 gene are known to be associated with hearing impairment^11^. The prevalence of some *GJB2* mutations differs considerably among ethnic groups. The 35delG mutation is the most common variant in European populations^12^^,^^13^. The carrier frequency of 35delG ranges between 0.97% and 2.24% in the southeastern region of Brazil^14^^,^^15^. In São Paulo state, the 35delG mutation was the most frequent (12.4%)^16^; it was found in 23% of the family cases, and 6.2% of the simplex cases. The 235delC mutation is predominant in Asian populations as a whole^17^, ^18^, ^19^, ^20^, ^21^, whereas the 167delT mutation is also frequent in Ashkenazi Jewish population^22^, ^23^, ^24^.

It is known that *GJB6* mutations are a common cause of deafness^25^. A deletion of 243Kb named del*GJB6*-D13S1830 mutation in *GJB6* gene is the second most frequent genetic cause of non-syndromic prelingual hearing impairment in the Spanish population^26^. It has also been described among Ashkenazi Jews^27^ and in French non-syndromic hearing loss patients^28^.

Some studies have reported the genetic frequency of *GJB2* mutations and del*GJB6*-D13S1830 mutation related with non-syndromic deafness in Brazilian populations^12^^,^^16^^,^^29^, ^30^, ^31^. North Brazilian populations are composed of a highly interethnic admixture with an added European gene contribution^32^ and no previous data are available for the allelic variants in *GJB2* or the frequency of the del*GJB6*-D13S1830 mutation in the Amazon region. In order to establish the prevalence of *GJB2* mutations among deafness patients in this region, we investigated 77 simplex cases of prelingual non-syndromic hearing impairment. Additionally, we investigated the prevalence of the del*GJB6*-D13S1830 deletion.

## METHOD

This study was approved by the Research Ethics Committee of João de Barros Barreto University Hospital (protocol n. 2241/05).

### Subjects and clinical evaluation

The study was conducted on seventy-seven children with prelingual non-syndromic hearing loss. All seventy-seven probands were unrelated and simplex cases of deafness. Samples were obtained from a School for the Deaf in Belém, Pará, Brazil. Syndromic patients were not included in this study. All the seventy-seven children were severe-to-profound prelingual hearing impairment cases. For each child, the complete medical history and questionnaires were administered to ensure that hearing loss was not the result of environmental causes: maternofetal infection, perinatal complications, meningitis, mumps, prenatal, prolonged use of antibiotics/drug ototoxicity and acoustic trauma. All children underwent an otoscopy, audiovestibular tests and available audiometry, and a general examination including systematic examination of syndromic form was evaluated. Written informed consent was obtained from all the patients' parents.

### Molecular Analysis

DNA was extracted from EDTA anticoagulated whole blood using the phenol-chloroform method and precipitation with ethanol. To identify GJB2 mutations, a DNA fragment containing the entire coding region was amplified using the primer pair in Polymerase Chain Reaction (PCR) and two additional internal primers were used in the DNA sequencing of Cx26: GJB2-1F (5′-GTGTTG-TGTGCATTCGTCTTTTC-3′) forward primer for PCR and sequencing; GJB2-2R (5′-CCTCATCCCTCTCATGCTGTC-TA-3′) reverse primer for PCR and sequencing; GJB2-4F (5′-GGAAGTTCATCAAGGGGGAGATA-3′) primer for internal forward sequencing, and GJB2-3R (5′-ACCTTC-TGGGTTTTGATCTCC TC-3′) primer for internal reverse sequencing.

PCR conditions were for 35 cycles with denatu-ration at 94°C for 1 min, annealing at 60°C for 1 min and extension at 72°C for 1 min. In all cases of *GJB2* gene analysis, bi-directional DNA sequencing was performed and in some cases internal primers were used to confirm the result. They were sequenced on an *ABI Prism Big-Dye Terminator Cycle Sequencing Kit*^TM^ *(Applied Biosystems)* and electrophoresed on an *ABI Prism 377 DNA Sequencer (Applied Biosystems)*. PCR amplification of delGJB6-D13S1830 mutation was performed by using primers and conditions previously described^26^.

## RESULTS

The results are summarized in Table 1. Analysis of the complete coding region of *GJB2* gene of 77 hearing-impaired children revealed four different mutations previously described: 35delG, M34T, V95M and V27I. Allele variants in *GJB2* gene were found in 40% of all cases (31 of the 77 patients). The most common mutation was V27I and detected in 22 patients: twenty individuals were heterozygous and two were homozygous. However, V27I mutation was previously reported to be a polymorphism without pathological effects and unrelated to hearing loss^17^. Pathogenic mutations were observed in 12% of patients. The 35delG mutation was detected in seven (9%) of the patients: one was homozygous and six patients were heterozygous, in which the second *GJB2* mutation was not found. The 35delG mutation accounted for 8 out of 10 GJB2 mutated pathogenic alleles. Two other missense mutations were observed, V95M and M34T, in two distinct heterozygous patients with the second allele being normal. In addition, screening for the del(GJB6-D13S1830) mutation was negative in all 77 patients.

**Table 1:** GJB2 mutations detected in Belém, state of Pará, Brazil.

Genotypes	No. of samples	(%)	Mutation type
35delG/35delG	1	1.3%	Frameshift
35delG/Wt	6	7.8%	
M34T/Wt	1	1.3%	Missense
V95M/Wt	1	1.3%	Missense
V27I/V27I	2	2.6%	Missense
V27I/Wt	20	26%	(Polymorphism)
Normal (Wt/Wt)	46	59.8%	
Total	77		

## DISCUSSION

This study is the first of its kind, describing the prevalence of *GJB2* and *GJB6* variants in Amazon population. Several studies have shown that the 35delG mutation is present in different ethnic and geographic groups, and accounted for up to 80% of *GJB2* mutations^33^. A previous study revealed a frequency of 2.1% for 35delG mutation in the northern region of Brazil^34^. Although our work was based on small numbers of patients, the results may confirm the importance of *GJB2* mutations in the etiology of hearing impairment in Belém - PA. This study shows that the 35delG mutation is the most common pathogenic *GJB2* allele. The 35delG mutant allele was found in 9% of the patients, which is in agreement with findings in previous studies of Brazilian population^29^^,^^30^^,^^35^. The pathological significance of the M34T mutation has been discussed. It was firstly associated with dominant hearing loss at DFNA3^8^. However, it has been reported by different authors as a polymorphism, a causative recessive or dominant mutation^8^^,^^36^, ^37^, ^38^, ^39^, ^40^. In a UK population study, the M34T mutation is in keeping with its classification as a mutation causing mild/moderate hearing loss in homozygosity or compound heterozygosity. This adds further evidence that M34T is a mild but functional variant with a larger effect in the 35delG group^41^. Another study of Finnish population support the hypothesis that the M34T is a pathogenic mutation and displays an autosomal recessive pattern of inheritance associated with mild to moderate non-syndromic sensorineural hearing impairment in the homozygous state^42^.

The M34T was reported in the South American population^43^ and Brazil^16^. The V95M mutation was observed in only one heterozygous patient. The missense mutation converts the amino acid valine to methionine at codon 95. Valine is invariant at this position in all known alpha and beta connexin genes^28^. V95M is reported to be much rarer^44^. It has been reported in one compound heterozygous patient with non-syndromic hearing impairment in a southeastern Brazilian population^29^. Of all *GJB2* mutations detected in our study, the V27I is the most frequent. This mutation is considered polymorphism and a non-pathological change related to hearing loss^17^^,^^22^. It is rarely observed in the North American population^22^. However, it is frequent among Asian populations, such as Chinese^45^; Japanese^17^; Korean^46^ and in Asian background population^47^. In a previous study among Brazilian patients, V27I mutation was found in two unrelated heterozygous individuals out of twenty-six sporadic cases^29^.

Given the high prevalence of V27I mutation, we raised the hypothesis that it resulted from the increased contribution of Amerindian genes in northern Brazilian populations^32^. To address the question, we investigated the frequency of the V27I mutation in a sample composed of 400 hearing individuals from Belém population. The results (unpublished data) demonstrated that the frequency of the V27I mutation among hearing individuals from Belém population is high (12.4%) and similar to the frequency of this mutation in the deaf patients investigated (15.5%). These frequencies are similar to those described in China^45^ and higher than in European populations. Thus, the Amerindian populations are probably the original source of the V27I mutation in the research sample.

del*GJB6*-D13S1830 mutation is the second most frequent genetic cause of non-syndromic prelingual hearing impairment in the Spanish population and a digenic pattern of inheritance involving *GJB2* mutations and del*GJB6*-D13S1830 mutation in *GJB6* gene is sug-gested^26^. Additionally, del(*GJB6*-D13S1830) allele was found in 7.1% of Brazilian deaf patients^26^. In another study, the del(*GJB6*-D13S1830) mutation was observed in only one compound heterozygous patient (35delG/ del(*GJB6*-D13S1830) from Brazil's southeastern region^30^.

In this work, the screening of 77 patients did not reveal this mutation and our results cannot suggest risk of hearing impairment due to del(*GJB6*-D13S1830) mutation in our population.

## CONCLUSION

*GJB2* mutated alleles were observed in 40% (31/77) of Amazonian deaf patients. However, the pathogenic variants (35delG, M34T and V95M) have been detected in 12% (9/77) of the cohort and are known to act in a recessive manner. Among nine patients with pathogenic mutations, eight carry only one mutation in the *GJB2* coding sequence, although the whole coding region of *GJB2* gene was sequenced in all samples by a bi-directional DNA sequencing, including the 35delG heterozygous. The putative second mutation in these patients could be located in non-analysed areas in *GJB2* gene such as splice sites. The hypothesis that the second mutation located in a second gene can not be ruled out.

The results of this pilot study should contribute to the development of the genetic diagnosis of deafness and could be important for public health issues, precise genetic counselling, and early treatment in this region. Moreover, further studies and research are needed to find out and analyse genes and variants to elucidate other genetic causes of hearing impairment in mixed populations.
